# Traffic Breakdown Probability Estimation for Mixed Flow of Autonomous Vehicles and Human Driven Vehicles

**DOI:** 10.3390/s23073486

**Published:** 2023-03-27

**Authors:** Lichen Su, Jing Wei, Xinwei Zhang, Weiwei Guo, Kai Zhang

**Affiliations:** 1 School of Automation Science and Electrical Engineering, Beihang University, Beijing 100191, China; 2 School of Electrical and Control Engineering, North China University of Technology, Beijing 100144, China; 3 Research Institute of Tsinghua, Pearl River Delta, Guangzhou 510530, China

**Keywords:** automated vehicles, human driven vehicles, traffic breakdown, queueing model, mixed traffic

## Abstract

Automated vehicles are expected to greatly boost traffic efficiency. However, how to estimate traffic breakdown probability for the mixed flow of autonomous vehicles and human driven vehicles around ramping areas remains to be answered. In this paper, we propose a stochastic temporal queueing model to reliably depict the queue dynamics of mixed traffic flow at ramping bottlenecks. The new model is a specified Newell’s car-following model that allows two kinds of vehicle velocities and first-in-first-out (FIFO) queueing behaviors. The jam queue join time is supposed to be a random variable for human driven vehicles but a constant for automated vehicles. Different from many known models, we check the occurrence of significant velocity drop along the road instead of examining the duration of the simulated jam queue so as to avoid drawing the wrong conclusions of traffic breakdown. Monte Carlo simulation results show that the generated breakdown probability curves for pure human driven vehicles agree well with empirical observations. Having noticed that various driving strategy of automated vehicles exist, we carry out further analysis to show that the chosen car-following strategy of automated vehicles characterizes the breakdown probabilities. Further tests indicate that when the penetration rate of automated vehicles is larger than 20%, the traffic breakdown probability curve of the mixed traffic will be noticeably shifted rightward, if an appropriate car-following strategy is applied. This indicates the potential benefit of automated vehicles in improving traffic efficiency.

## 1. Introduction

From the operation viewpoint, traffic breakdown refers to a sudden and significant traffic velocity drop that usually results in congestion. To maintain traffic efficiency, increasing numbers of researchers have shown interest in controlling traffic breakdowns during the last two decades [1,2,3,4]. However, the stochastic nature of traffic breakdown and its sensitiveness to environmental factors still prevent us from finding the exact occurring conditions and duration.

Traditionally, the term “capacity” has been frequently used to indicate the maximum flow rate that can pass through a section of road. However, many observations have now proven that, even at the same location, traffic breakdown does not necessarily occur at the same prevailing inflow level [5,6,7]. Therefore, more researchers and engineers use the term “pre-breakdown flow rate”, which refers to the flow rate observed immediately before traffic breaks down and investigate its relationship with traffic breakdown probability.

Empirical investigations reveal that this relationship can be depicted as an S-type monotonically increasing curve in the breakdown probability versus pre-breakdown flow rate plot. In many reports [8,9], the cumulative distribution function (CDF) for Weibull distribution is further used to fit this curve as:

(1) $$W(q)=1-\exp\left\{-{\left(\frac{q}{\alpha }\right)}^{\beta }\right\},$$

where *q* denotes the pre-breakdown flow rate, α>0 is the scale parameter, and β>0 is the shape parameter.

The origins of these S-type breakdown probability curves have attracted growing concern nowadays. Various dynamic models that have been proposed account for the instability of traffic flows under perturbations and the resulting traffic breakdowns. One promising research direction is investigating the evolution processes of the local jam queue and studying its links with expanded traffic flow congestion; e.g., [10,11]. The imperative benefit of such approaches is that we can estimate traffic breakdown probability based on vehicular trajectories data collected in practice.

Another important research direction is to take the size variation of a jam queue as a diffusion process and approximately obtain the solution of the associate master equation [12,13,14,15]. One major benefit of such approaches lies in the conciseness of the proposed diffusion model, as only the temporal (size) evolution of the jam queue needs to be analyzed. Based on Occam’s Razor, the simplest models might give the best explanation, if they can reproduce the breakdown phenomena.

Tests show that the above two kinds of approaches can yield S-type breakdown probability curves. These agreements indicate that both approaches capture an essential part of the stochastic nature for traffic breakdown. However, most proposed models have emphasized either the jam wave propagation properties [16] or the size evolution features of the jam queue [12,13]. This simplification may sometimes lead to wrong deductions; see examples presented in Section 2.1 below.

A question naturally arises: Need we consider both the temporal (size) and spatial (extent) features of the jam queue simultaneously? Without a thorough examination on the evolution processes of jam queues, we may not build a convincing bridge that links perturbation growth/dissipation and traffic breakdown phenomena. To solve this problem, we propose a stochastic temporal–spatial queueing model to interpret the traffic breakdown mechanism in [17]. Testing results indicated that temporal (size) evolution rather than spatial (extent) evolution of a jam queue dominates the stochastic nature of traffic breakdown around ramping bottlenecks. Therefore, we can resort to a temporal queueing model to characterize traffic breakdown probability.

In this paper, we consider the possible influence of recently emerging automated vehicles to traffic breakdown probability [18,19]. Usually, researchers find that shorter car-following gaps between automated vehicles at the microscopic level could directly lead to larger capacity and lower breakdown probability at the macroscopic level. To further examine the relationship between the penetration rate and the traffic breakdown probability, we modify the stochastic temporal queueing model proposed in [17] to reliably depict queue dynamics of mixed traffic flow at ramping bottlenecks. 

Moreover, it must be noticed that different automation levels of automated vehicles exist. In addition, different automated vehicles may adopt various car-following strategies [18,20]. As a result, the jam queue entering behavior and leaving behavior of automated vehicles may differ significantly. This difference may greatly change the breakdown probability of mixed traffic flow. Therefore, we first establish the relationship between the car-following strategy of automated vehicles and the corresponding jam queue entering/leaving behavior, and then study their influence on the breakdown probability.

We carried out hundreds of simulations to examine the testing result. As expected, the Monte Carlo simulation results indicate that when the penetration rate of automated vehicles is larger than 20%, the traffic breakdown probability curve of the mixed traffic will be noticeably shifted rightward. This indicates the potential benefit of automated vehicles in improving traffic efficiency.

To give a detailed explanation of our approach, the rest of this paper is organized as follows: Section 2 first briefly reviews some background of this study and then carefully introduces the temporal queueing model. How to calculate traffic breakdown probability for mixed traffic flow via this queueing model is also presented. We give prominence to the influence of the various car-following behaviors of automated vehicles. Section 3 executes thousands of rounds of Monte Carlo simulations and compares the simulated traffic breakdown probability curves with the Weibull CDF traffic breakdown curves. Finally, Section 4 makes conclusions on our findings.

## 2. A Stochastic Temporal Queueing Model for Traffic Breakdown Probability

### 2.1. Some Background

Most dynamic explanations on traffic breakdown rely on appropriate modeling of the jam queue evolution [21]. The common idea behind these approaches can be summarized as follows.

**Figure 1:**
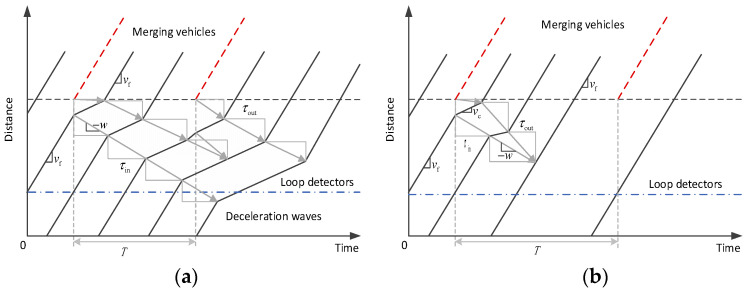
The growth and dissipation of a jam queue. (**a**) Jam queue growth that results in a traffic breakdown; (**b**) Jam queue dissipation that results in smooth traffic.

When a small jam queue (congested vehicle cluster) emerges, two competing processes become active simultaneously. On one hand, the upstream vehicles will continually join this jam queue and makes it increase in size; on the other hand, the downstream vehicles will continually leave this jam queue and make it decrease in size. Therefore, the growth/dissipation of a jam queue is controlled by the rate of vehicles that join in/depart from it. If the joining rate is on average larger than the departing rate, a small localized and temporary jam queue caused by perturbation can be magnified into a wide jam (see Figure 1a); otherwise, it will normally dissipate (see Figure 1b).

Let us focus on the ramping perturbation scenarios. If the jam queue caused by the previously merging vehicle does not dissipate before the next merging vehicle joins the main road, we may see multiple jam queues collide and merge into a wide jam that propagates upstream and causes congestion around the loop detector. As a result, a traffic breakdown is detected according to the collected data.

Various models have thus been proposed to describe the stochastic behaviors and outcomes of these two competing processes. For example, Kim and Zhang [10,16] and Son et al. [11] simulated traffic breakdown phenomena based upon Newell’s simplified model. Their work set up a bridge between the macroscopic traffic breakdown phenomenon and the microscopic car-following behaviors. Results in Son et al. [11] yielded an S-type curve breakdown probability curve. However, the ac/deceleration wave speeds were not well examined in these studies and the obtained breakdown probability curves were thus not smooth or steep enough.

Another important series of studies took jam queue evolution as nucleation processes [12,13,14,15,22,23]. More precisely, the jam queue growth was modeled as a one-dimensional diffusion process. Solving the corresponding Fokker–Planck equation, we can obtain a smooth S-type breakdown probability curve. Another approach was proposed in [17], which used a queueing model to describe the evolution of the jam queue and thus the breakdown probability. However, all these approaches focus on the temporal evolution of the jam queue and neglect its spatial evolution. Whether we should also consider the spatial patterns of jam queues remains to be answered.

In summary, the S-type breakdown probability curves attained in these previous studies indicate that these temporal queueing evolution models had captured the stochastic nature of traffic breakdown [8,24,25,26]. However, in some situations, the temporal evolution process does not fully characterize the dynamics of the jam queue.

**Figure 2:**
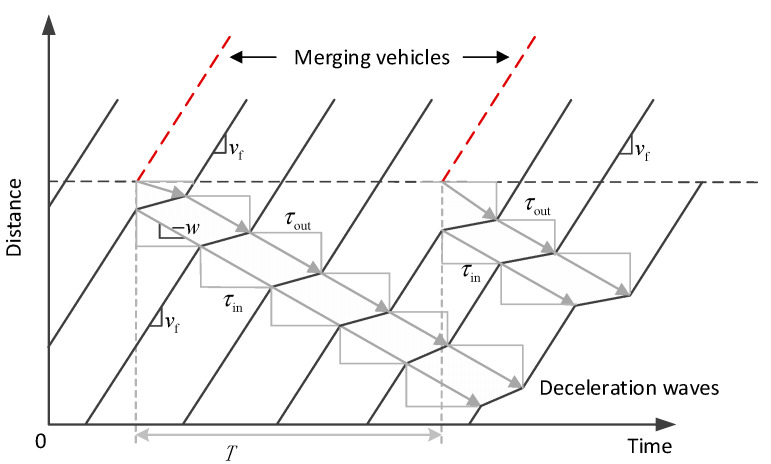
An illustration of moving localized cluster (MLC), in which the jam queue will not dissipate in a short time, but no breakdown will be generated.

The so-called moving localized cluster (MLC) is a typical example (see Figure 2). As pointed out in [27,28], under some conditions, the size of a jam queue may be kept unchanged for a relatively long time but its location will propagate upstream. However, no significant congestion will be generated.

### 2.2. A Deterministic Temporal–Spatial Queueing Model

In order to better describe the models, let us recall another queueing model that has been proposed very recently. Aiming to quantitatively analyze congestion patterns observed around on/off-ramps, a deterministic temporal–spatial queueing model was proposed very recently in [29]. It can be viewed as a specialized Newell’s model [30] that addresses the scenario of jam queue formations, propagations, and dissipations.

To adopt a small as possible set of parameters and rules, this queueing model assumes that all the vehicle trajectories can be simplified as piecewise linear. Moreover, there are only two kinds of vehicle speed: free flow speed, $v_{f}$, and congested flow speed, $v_{c}$. Thus, we only have two kinds of slopes for all the trajectories.

**Figure 3:**
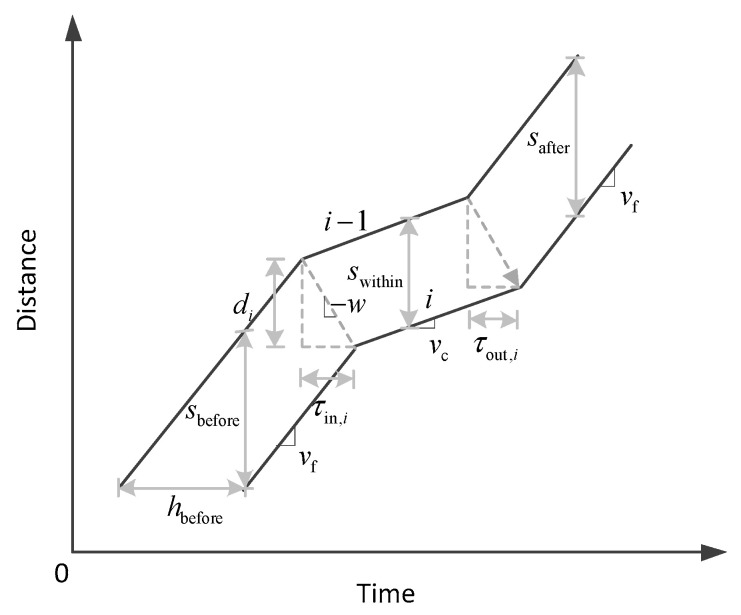
An illustration of the deterministic temporal–spatial queueing model.

The kernel mechanism of this queueing model is illustrated in Figure 3. Suppose the join time and departing time for the ith vehicle are denoted as $\tau_{\mathrm{in},i}$ and $\tau_{\mathrm{out},i}$, respectively. If the propagation speed of the upstream shock front (congestion wave) is constant w, the change in spacing between two consecutive vehicles through a jam queue can be written as [29]:

(2) $$\left\{\begin{array}{l} d_{i}=w\tau_{\mathrm{in},i}\\ s_{\mathrm{before}}=(v_{f}+w)\tau_{\mathrm{in},i}\;\\ s_{\mathrm{within}}=(v_{c}+w)\tau_{\mathrm{in},i}\\ s_{\mathrm{after}}=(v_{c}+w)\tau_{\mathrm{in},i}+(v_{f}-v_{c})\tau_{\mathrm{out},i} \end{array}\right.,$$

Theoretical analysis and numerical simulations show that this simple model provides an acceptable description of car-following dynamics and establishes a reasonable starting point for modeling more complex traffic phenomena. One benefit of this model is that we can reveal the implicit coupling relations between the main road flow rate and vehicle headway/spacing, as well as their dependence on jam queue join time; see discussions in [29].

However, this deterministic temporal–spatial queueing model does not consider the stochastic features of jam queue join time and jam wave speed in reality. Thus, it cannot reproduce the stochastic properties of traffic flows, e.g., breakdown probability.

### 2.3. Possible Influence of Car-Following Styles of Automated Vehicles

As is well known, various automation levels of automated vehicles now exist. This is partly because many drivers wish to retain the will to drive fully or partially autonomously, and the current level of development of artificial intelligence means that it is still difficult to achieve fully driverless vehicles in complex traffic environments. In addition, relevant laws and regulations are still being further developed and improved.

As a result, the Society of Automotive Engineers (SAE) released a six-level classification principle for intelligent vehicle autonomy in 2016 [31]. China’s Ministry of Industry and Information Technology (MIIT) in 2022 also released a similar recommended national standard for “Vehicle Driving Automation Classification” divided into six levels, of which Level 0 is emergency assistance; Level 1 is partial driving assistance, where the driving automation system is able to perform vehicle lateral or longitudinal motion control continuously within its designed operating conditions; and Level 2 is combined driving assistance, where in addition to the above functions, it also has partial target and event detection and response capabilities. Automated vehicles in Levels 3 to 5 will be able to maintain a closer car-following gap than human drivers. However, automated vehicles in Levels 0 to 2 will still need a relatively larger car-following gap than human drivers.

In recent years, vehicular communication techniques have greatly improved. Vehicles equipped with vehicle-to-everything (V2X) communication equipment can acquire the information not just on the vehicle immediately in front (through on-board sensors) but also on the leading vehicle or vehicles further in front [32,33,34]. The availability of precise position and speed information of neighboring vehicles allows automated vehicles to keep an even shorter car-following gap [35].

Summarizing all the related models, we analyze three kinds of automated vehicle car-following strategies in this paper.

The first kind of automated vehicle car-following strategy adopts a constant spacing between its leading vehicles, and this spacing is shorter than the average spacing maintained by human drivers. The automated vehicle platoons assisted by V2X communication adopt such a car-following strategy [36].

The second kind of automated vehicle car-following strategy adopts a constant spacing between its leading vehicles, and this spacing is larger than the average spacing maintained by human drivers. Automated vehicles in Levels 1 and 2 adopt such a car-following strategy.

The third kind of automated vehicle car-following strategy adopts a constant time headway between its leading vehicles, and this time headway is equal to the average time headway maintained by human drivers. Automated vehicles in Levels 3 to 5 adopt such a car-following strategy, as they usually imitate the driving behaviors of human drivers.

### 2.4. A Stochastic Temporal Queueing Model for Mixed Traffic

Indeed, we find that the jam queue increases when a vehicle joins the jam queue, while the jam queue decreases when a vehicle accelerates and leaves this jam queue. Therefore, the rate of vehicles that join in/depart from the jam queue characterizes the birth/death of the jam queue. If the joining rate is on average smaller than the departing rate, a small perturbation will finally dissipate. Otherwise, a small perturbation will eventually grow into a wide jam.

This assumption leads to a simple queueing model for the mixed traffic of both automated vehicles and human driven vehicles based on Newell’s simplified car-following model. The average joining rates are governed by the upstream inflow rate, while the departing rates are assumed to be a normally distributed random variable. In addition, vehicle headway changes with the upstream inflow rate and thus influences the joining time. According to our previous studies [37,38], vehicle headway is a random variable that belongs to shifted log-normal distributions with a scaling parameter. Such right-skew distributed random variables finally generate the S-type traffic breakdown probability curve.

To better describe the microscopic stochastic car-following behaviors and their influence on macroscopic traffic flow instability, we modify the above queueing model in three ways:

(1) We assume the jam queue join time for human driven vehicles is a random variable whose distribution changes with the main road flow rate;
(2) We assume the jam queue join time for automated vehicles is a determinant variable whose value linearly changes with the main road flow rate; and different types of automated vehicles will have different spacing policies;
(3) We assume the jam wave speeds for both automated vehicles and human driven vehicles are the same constant, as automated vehicles need to mimic the preferred car-following behaviors of human drivers.

All these assumptions are made according to empirical observations. In the following simulation tests, the parameters of these distributions will be determined from empirical data, too.

Similar to [17], we set the spacing $s_{\mathrm{before}}^{\left(\mathrm{human}\right)}$ of human driven vehicles coming from upstream as:

(3) $$S_{\mathrm{before}}^{\left(\mathrm{human}\right)}-S_{0}\;\sim \;Log_{N}\left(\mu_{s,}\sigma_{s}^{2}\right),$$

where $s_{0}$ is the infimum of spacing and $\mu_{s}$ and $\sigma_{s}$ are the corresponding location and scale parameters, respectively.

Differently, for automated vehicles, we set the spacing $s_{\mathrm{before}}^{\left(\mathrm{auto}\right)}$ of automated vehicles coming from upstream as:

(4) $$S_{\mathrm{before}}^{\left(\mathrm{auto}\right)}=S_{0}+\exp\left(\mu_{s}+\sigma_{s}^{2}/2\right),\;S_{\mathrm{before}}^{\left(\mathrm{auto},1\right)}<E\left[S_{\mathrm{before}}^{\left(\mathrm{human}\right)}\right],\;S_{\mathrm{before}}^{\left(\mathrm{auto},2\right)}>E\left[S_{\mathrm{before}}^{\left(\mathrm{human}\right)}\right],\;S_{\mathrm{before}}^{\left(\mathrm{auto},3\right)}=\frac{v_{\mathrm{free}}^{\mathrm{auto}}}{v_{\mathrm{free}}^{\mathrm{human}}}E\left[S_{\mathrm{before}}^{\left(\mathrm{human}\right)}\right]=E\left[S_{\mathrm{before}}^{\left(\mathrm{human}\right)}\right],$$

where $s_{\mathrm{before}}^{\left(\mathrm{auto},1\right)}$, $s_{\mathrm{before}}^{\left(\mathrm{auto},1\right)}$, and $s_{\mathrm{before}}^{\left(\mathrm{auto},1\right)}$ are the constant spacing of the three kinds of automated vehicle car-following strategies. Here, we assume that the free speeds of the human driven vehicles and the automated vehicles are equal, i.e., $v_{\mathrm{free}}^{\mathrm{human}}=v_{\mathrm{free}}^{\mathrm{auto}}=v_{f}$.

From Equation (2), we have the joint times of human driven vehicles and automated vehicles into a jam queue formulated as:

(5) $$\tau_{\mathrm{in}}^{\left(\mathrm{human}\right)}=\frac{s_{\mathrm{before}}^{\left(\mathrm{human}\right)}}{v_{f}+w},$$

(6) $$\tau_{\mathrm{in}}^{\left(\mathrm{auto}\right)}=\frac{s_{\mathrm{before}}^{\left(\mathrm{auto}\right)}}{v_{f}+w},$$

Further, assuming that the mixed ratio between automated vehicles and human driven vehicles is p, we have the mixed upstream vehicle spacing and flow rate formulated as:

(7) $$s_{\mathrm{before}}^{\left(\mathrm{mix}\right)}=ps_{\mathrm{before}}^{\left(\mathrm{human}\right)}+\left(1-p\right)s_{\mathrm{before}}^{\left(\mathrm{auto}\right)},$$

(8) $$q_{\mathrm{before}}^{\left(\mathrm{mix}\right)}=q_{\mathrm{before}}^{\left(\mathrm{human}\right)}+q_{\mathrm{before}}^{\left(\mathrm{auto}\right)}=\frac{v_{f}}{ps_{\mathrm{before}}^{\left(\mathrm{human}\right)}+\left(1-p\right)s_{\mathrm{before}}^{\left(\mathrm{auto}\right)}},$$

For human driven vehicles, the expectations of spacing $E[s_{i}]$ and the corresponding inflow rate $q_{\mathrm{before}}^{\left(\mathrm{mix}\right)}$ yields:

(9) $$q_{\mathrm{before}}^{\left(\mathrm{mix}\right)}=\frac{v_{f}}{pE[s_{\mathrm{before}}^{\left(\mathrm{human}\right)}]+\left(1-p\right)s_{\mathrm{before}}^{\left(\mathrm{auto}\right)}},$$

We can get the relationship between the average join time $\mu_{\tau }$ and the inflow rate $q_{\mathrm{before}}^{\left(\mathrm{mix}\right)}$ as:

(10) $$q_{\mathrm{before}}^{\left(\mathrm{mix}\right)}=\left(\frac{1}{pE[\tau_{\mathrm{in}}^{\left(\mathrm{human}\right)}]\left(v_{f}+w\right)+\left(1-p\right)s_{\mathrm{before}}^{\left(\mathrm{auto}\right)}}\right)v_{f},$$

(11) $$\mu_{\tau }=\log\left(\frac{v_{f}}{pq_{\mathrm{before}}^{\left(\mathrm{mix}\right)}(v_{f}+w)}-\frac{1}{p}\tau_{0}\right)-\frac{\delta_{\tau }{}^{2}}{2},$$

Based on this relationship, we can further simulate the time evolution of a jam queue, when the mixed ratio p, the upstream inflow rate q, the free flow speed $v_{f}$ and the propagation speed w of the upstream shock front (congestion wave), the infimum of spacing $s_{0}$, the location parameter $\mu_{\tau }$, and the scale parameter $\sigma_{\tau }$ of the human driven vehicles’ spacing characteristics are given.

## 3. Results

### 3.1. The Monte Carlo Simulation Procedure

In order to calculate traffic breakdown probability with respect to pre-breakdown flow rates, we resort to Monte Carlo simulations [39,40].

More precisely, we will simulate to check whether a newly formed jam queue could dissipate in a given time period T.

The number of approaching vehicles within a time period T is about $\hat{n}=\left\lfloor q_{S}T\right\rfloor$, where $\left\lfloor z\right\rfloor$ denotes the largest integer not greater than z. We do not need to simulate for a long time period T that is larger than 60 s [17]. This further reduces our simulation time cost.

**Figure 4:**
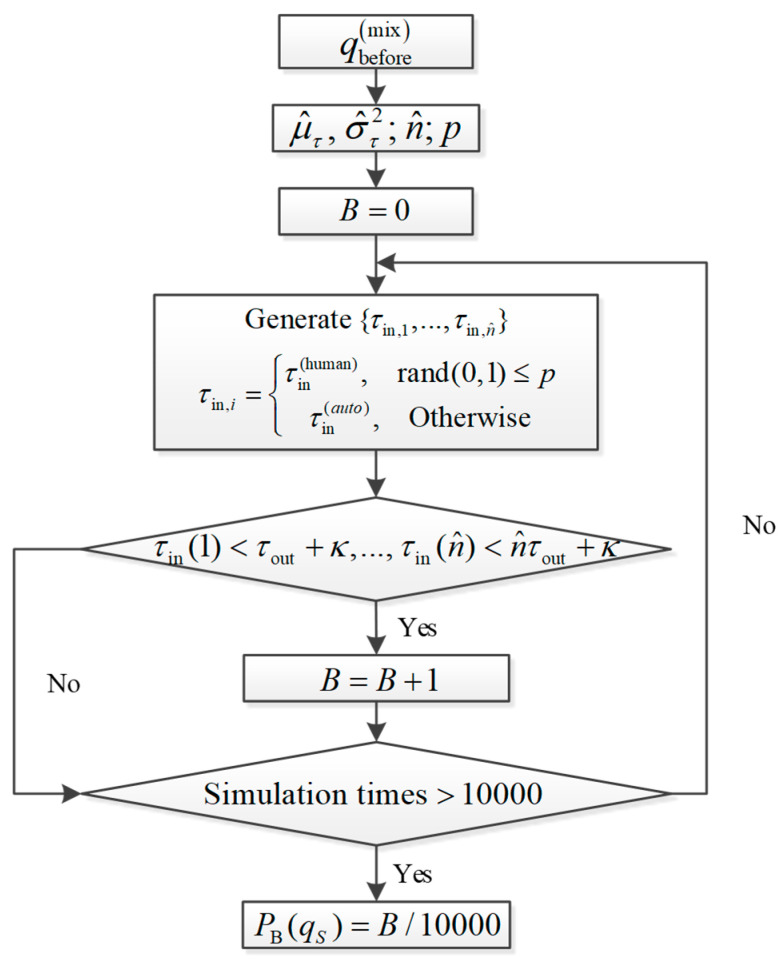
Estimation approach of breakdown probability by Monte Carlo simulation.

The basic simulation outflow is shown in Figure 4, which includes the following steps:

Step 0: Given the upstream inflow $q_{\mathrm{before}}^{\left(\mathrm{mix}\right)}$, $\tau_{\mathrm{out}}$, the mixed ratio p, and a long enough time window T, initialize the number of traffic breakdowns B=0.

Step 1: Generate $\hat{n}$ independent and identically distributed (i.i.d.) random variables $\left\{\tau_{\mathrm{in},1},\ldots ,\tau_{\mathrm{in},\hat{n}}\right\}$. The jam queue join time $\tau_{\mathrm{in}}^{\left(\mathrm{human}\right)}$ of human driven vehicles follows the log-normal distribution with (${\hat{\mu }}_{\tau }$,$\sigma_{\tau }^{2}$). According to Equations (4) and (6), the constant jam queue join time $\tau_{\mathrm{in}}^{\left(\mathrm{auto}\right)}$ of automated vehicles is equal to the average join time of human driven vehicles, i.e.,:

(12) $$\tau_{\mathrm{before}}^{\left(\mathrm{auto}\right)}=\exp\left(\mu_{\tau }+{\delta_{\tau }}^{2}/2\right)+\tau_{0},\;\tau_{\mathrm{before}}^{\left(\mathrm{auto},1\right)}=\frac{s_{\mathrm{before}}^{\left(\mathrm{auto},1\right)}}{v_{f}+w}<E\left[\tau_{\mathrm{in}}^{\left(\mathrm{human}\right)}\right]\;\tau_{\mathrm{before}}^{\left(\mathrm{auto},1\right)}=\frac{s_{\mathrm{before}}^{\left(\mathrm{auto},2\right)}}{v_{f}+w}>E\left[\tau_{\mathrm{in}}^{\left(\mathrm{human}\right)}\right]\;\tau_{\mathrm{before}}^{\left(\mathrm{auto},3\right)}=\frac{s_{\mathrm{before}}^{\left(\mathrm{auto},3\right)}}{v_{f}+w}=E\left[\tau_{\mathrm{in}}^{\left(\mathrm{human}\right)}\right]$$

Then, check whether the breakdown criteria are satisfied; if yes, let B=B+1.

Step 2: Repeat Step 1 for 10,000 times.

Step 3: Calculate the simulated breakdown probability as $P_{B}(q_{\mathrm{before}}^{\left(\mathrm{mix}\right)})=B/10000$. Choose another value of $q_{\mathrm{before}}^{\left(\mathrm{mix}\right)}$ and repeat the whole procedure until we obtain the entire traffic breakdown curve.

To automatically detect congestion formed in simulation, we assume that traffic is unqueued (uncongested or free flowing) if no significant velocity drop is detected upstream of that location. Conversely, if changes in velocity are felt upstream after we introduce a disturbance at a location, we say that traffic is queued (or congested). This assumption is natural and had been widely used in practice [21].

### 3.2. Parameter Settings

**Figure 5:**
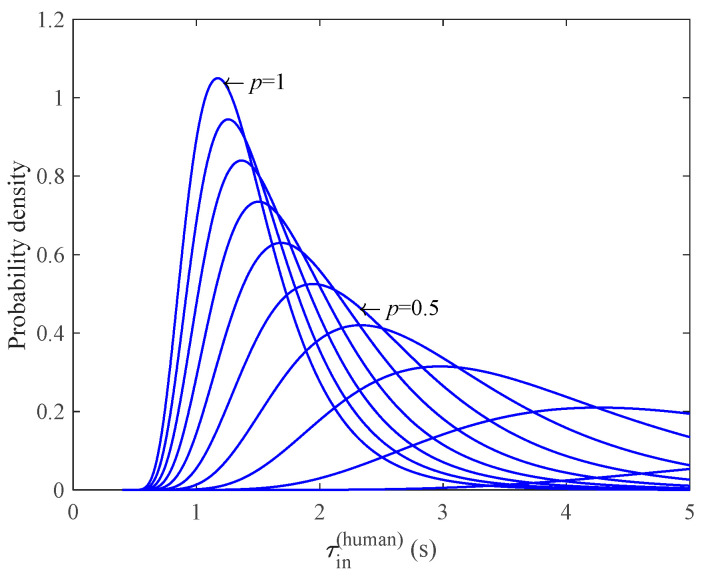
The estimated probability density functions of human driven vehicles $\tau_{\mathrm{in}}^{\left(\mathrm{human}\right)}$ in terms of mixed ratio p, where $q_{\mathrm{before}}^{\left(\mathrm{mix}\right)}$ = 2000 veh/h.

**Figure 6:**
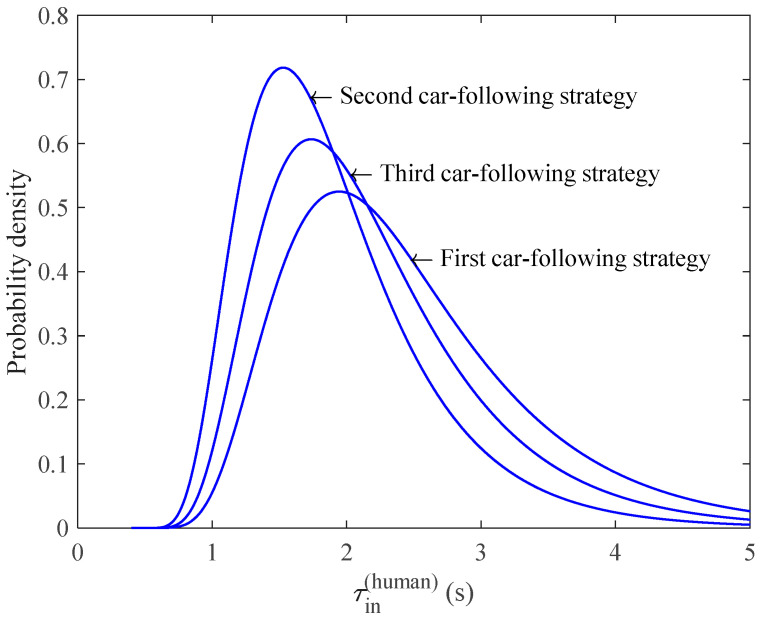
The estimated probability density functions of human driven vehicles $\tau_{\mathrm{in}}^{\left(\mathrm{human}\right)}$ in terms of the kinds of automated vehicle car-following strategies, where $q_{\mathrm{before}}^{\left(\mathrm{mix}\right)}$ = 2000 veh/h and p=0.6.

Following the statistical results from traffic data [38], we set $\sigma_{\tau }=0.446$, the free flow speed $v_{f}=20\;m/s$, and the propagation speed w=5 m/s of the congestion wave. Thus, for a given mixed ratio p, the probability density function (PDF) of $\tau_{\mathrm{in}}^{\left(\mathrm{human}\right)}$ could be estimated from Equation (11), as illustrated in Figure 5. The horizontal coordinates of Figure 5 are time and the vertical coordinates are probability densities, and the different curves indicate the estimated breakdown probabilities at different autonomous vehicle penetration rates. In addition, with the same arrival rate $q_{\mathrm{before}}^{\left(\mathrm{mix}\right)}$ and mixed ratio p, different car-following strategies will also affect the probability density distribution of $\tau_{\mathrm{in}}^{\left(\mathrm{human}\right)}$, as illustrated in Figure 6.

### 3.3. Simulation Results

**Figure 7:**
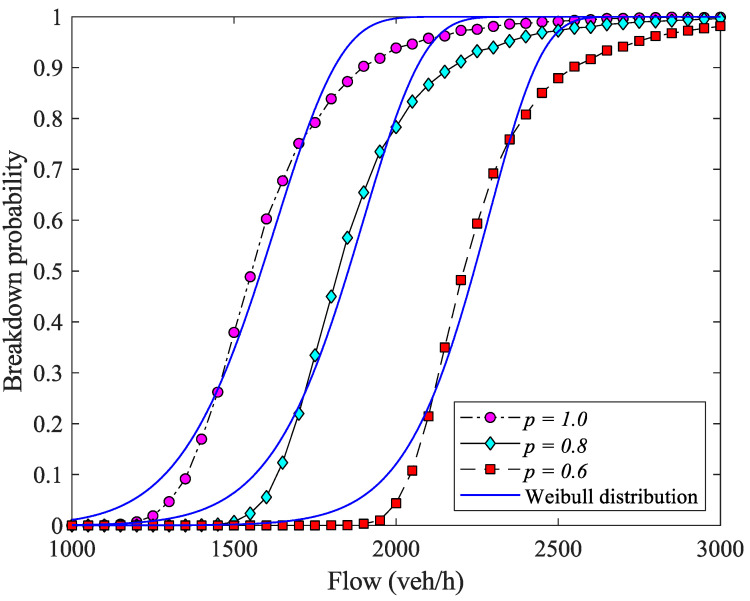
Comparisons between the G/D/1 and the fitting Weibull distributions in terms of mixed ratio p, given $\tau_{\mathrm{out}}=2s$.

In simulations, we increase $q_{s}$ from 1000 veh/h to 3000 veh/h with a step of 50 veh/h to get the breakdown probability curve. Figure 6 and Figure 7 show the simulation results with different mixed ratio p.

In order to compare the simulation results with the widely used Weibull CDF curve, we use the least-squares criteria to obtain the Weibull CDF curve that best fits the simulated traffic breakdown probability curve:

(13) $$R(\alpha ,\beta )=\sum_{q_{S}}{\left(W(q_{S}|\alpha ,\beta )-P_{B}(q_{S})\right)}^{2}$$

where R(α,β) is the square residuals between the breakdown probability curve $P_{B}(q_{S})$ and the Weibull distribution with parameters of α,β. The fitting parameters can be obtained as:

(14) $$\hat{\alpha },\hat{\beta }=\arg\underset{\alpha ,\beta }{\min}R(\alpha ,\beta ),$$

then, the least-squares residual is:

(15) $$\mathrm{LSR}=\sum_{q_{S}}{\left(W(q_{S}|\hat{\alpha },\hat{\beta })-P_{B}(q_{S})\right)}^{2},$$

Figure 7 shows that with different mixed ratio p, the differences between our simulation results and the least-square fitting results of the Weibull CDF curve are quite small. It suggests that the proposed queueing model can capture the non-decreasing sigmoid breakdown probability in mixed traffic flow. Moreover, when the ratio of human driving vehicles decreases, the traffic breakdown probability curve moves rightward, indicating that automated vehicles can effectively reduce the breakdown probability under the same $q_{\mathrm{before}}^{\left(\mathrm{mix}\right)}$.

**Figure 8:**
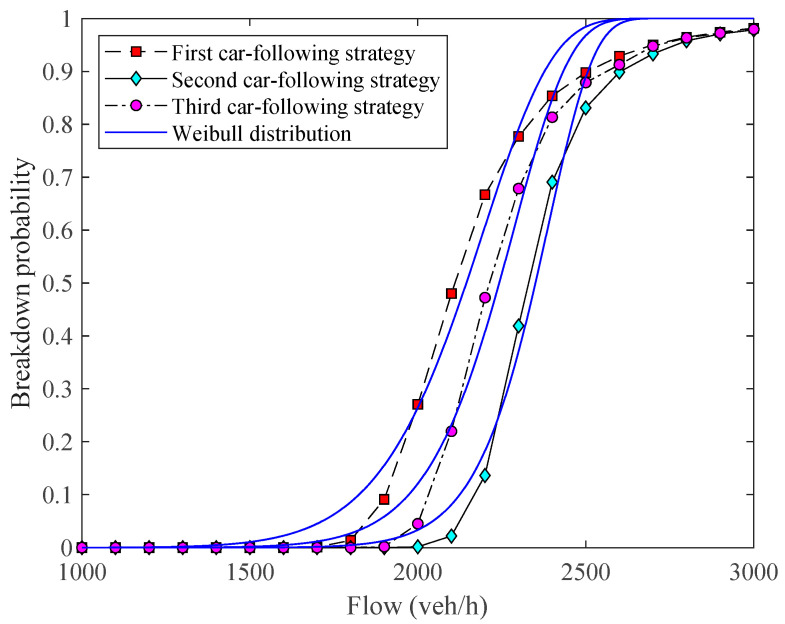
Comparisons between the G/D/1 and the fitting Weibull distributions in terms of automated vehicle car-following strategies, given $\tau_{\mathrm{out}}=2s$ and p=0.6.

Figure 8 shows that with the different car-following strategies, the difference between our simulation results and the least-square fitting results of the Weibull CDF curve is also small. The results show that when the car-following spacing increases, the probability curve moves to the right. It indicates that increasing the car-following spacing can effectively reduce the breakdown probability, that is, automated vehicles in Levels 1 and 2 have a lower breakdown probability.

**Figure 9:**
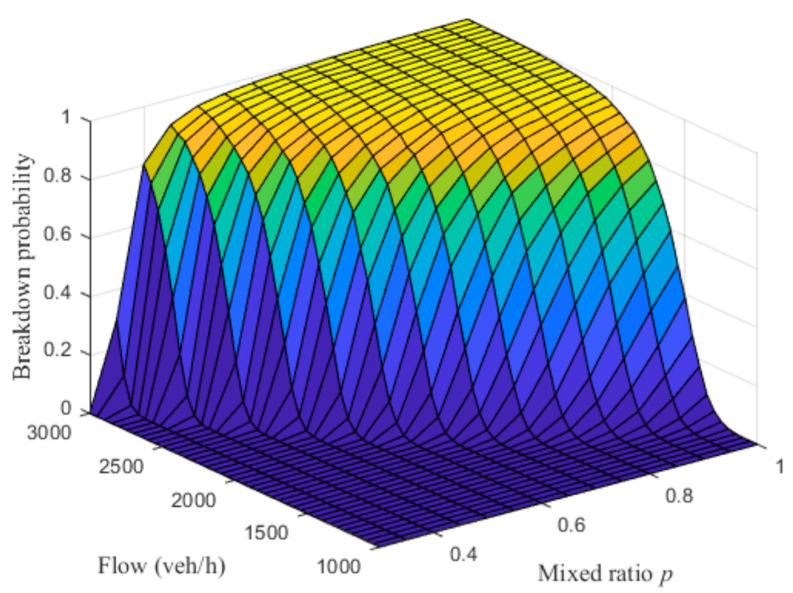
Breakdown probability with different mixed ratio p.

Figure 9 shows the simulation results of breakdown probability with different mixed ratios. The darker the color represents the smaller the z-axis value, as shown in Figure 9, blue represents the value of 0, yellow represents of 1. The results show that with the decrease in the ratio of human driving vehicles, i.e., the penetration rate of automated vehicles increases, the breakdown probability will decrease. For a certain flow, when the maxed ratio decreases to a certain value, the breakdown probability will decrease significantly. For example, when the mixed flow is 2000 veh/h, traffic with all human driving vehicles (p = 1) has a high breakdown probability (0.93). However, when the ratio of human driving vehicles decreases to p = 0.8, the breakdown probability will decrease rapidly. When further reduced to p = 0.6, the breakdown probability reaches a lower range (less than 0.11).

**Figure 10:**
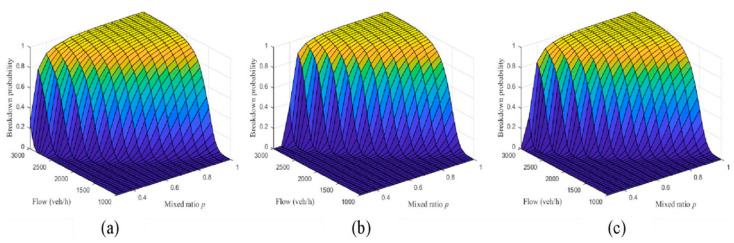
Breakdown probability with automated vehicle car-following strategies: (**a**) first kind of car-following strategy; (**b**) second kind of car-following strategy; (**c**) third kind of car-following strategy.

Figure 10 compares the simulation results of breakdown probability with different automated vehicle car-following strategies. The darker the color represents the smaller the z-axis value, as shown in Figure 10, blue represents the value of 0, yellow represents of 1. The results show that the second car-following strategy has a smaller breakdown probability under the same arrival rate and the proportion of human driven vehicles. This indicates that automated vehicles in Levels 1 and 2 have a lower breakdown probability. This is contrary to the common intuition of researchers. Therefore, from the perspective of macroscopic traffic system control, shortening the car-following spacing of automated vehicles does not necessarily improve traffic efficiency, but it will increase the breakdown probability.

## 4. Conclusions

Automated vehicles are widely considered to improve road safety, increase road capacity, and stabilize traffic. However, it is widely accepted that autonomous vehicles can stabilize traffic flow, but little consideration is given to the traffic environment in which the vehicles themselves operate, i.e., little research has been done on the contribution of autonomous vehicles to stabilizing traffic during traffic breakdown. In this paper, we modify a classical jam queue model to describe mixed traffic flow and study the possibly resulting traffic breakdown phenomena initialized by small perturbation. Testing results indicate that this new model captures some major characteristics of jam queue evolution for mixed traffic and is able to predict the variation of breakdown probabilities with respect to the penetration rate of automated vehicles. Moreover, we compare the three kinds of automated vehicle car-following strategies. The results show that a shorter car-following spacing will increase the breakdown probability, while automated vehicles in Levels 1 and 2 can reduce the breakdown probability due to the larger car-following spacing. Therefore, we suggest that automated vehicles need to adjust their car-following strategy by choosing a car-following strategy with shorter spacing when there is no ramp and switching to a car-following strategy with larger spacing near the ramp to reduce the breakdown probability.

## Data Availability

Not applicable.
